# Giant liver hemangioma resected by trisectorectomy after efficient volume reduction by transcatheter arterial embolization: a case report

**DOI:** 10.1186/1752-1947-4-283

**Published:** 2010-08-23

**Authors:** Nobuhisa Akamatsu, Yasuhiko Sugawara, Masahiko Komagome, Takashi Ishida, Nobuhiro Shin, Narihiro Cho, Fumiaki Ozawa, Daijo Hashimoto

**Affiliations:** 1 Department of Hepato-biliary-pancreatic Surgery, Saitama Medical Center, Saitama Medical University, 1981 Tsujido-cho, Kamoda, Kawagoe, Saitama 350-8550, Japan; 2 Artificial Organ and Transplantation Division, Department of Surgery, Graduate School of Medicine, University of Tokyo, 7-3-1 Hongo, Bunkyo-ku, Tokyo 113-8655, Japan

## Abstract

**Introduction:**

Liver hemangiomas are the most common benign liver tumors, usually small in size and requiring no treatment. Giant hemangiomas complicated with consumptive coagulopathy (Kasabach-Merritt syndrome) or causing severe incapacitating symptoms, however, are generally considered an absolute indication for surgical resection. Here, we present the case of a giant hemangioma, which was, to the best of our knowledge, one of the largest ever reported.

**Case presentation:**

A 38-year-old Asian man was referred to our hospital with complaints of severe abdominal distension and pancytopenia. Examinations at the first visit revealed a right liver hemangioma occupying the abdominal cavity, protruding into the right diaphragm up to the right thoracic cavity and extending down to the pelvic cavity, with a maximum diameter of 43 cm, complicated with "asymptomatic" Kasabach-Merritt syndrome. Based on the tumor size and the anatomic relationship between the tumor and hepatic vena cava, primary resection seemed difficult and dangerous, leading us to first perform transcatheter arterial embolization to reduce the tumor volume and to ensure the safety of future resection. The tumor volume was significantly decreased by two successive transcatheter arterial embolizations, and a conventional right trisectorectomy was then performed without difficulty to resect the tumor.

**Conclusions:**

To date, there have been several reports of aggressive surgical treatments, including extra-corporeal hepatic resection and liver transplantation, for huge hemangiomas like the present case, but because of its benign nature, every effort should be made to avoid life-threatening surgical stress for patients. Our experience demonstrates that a pre-operative arterial embolization may effectively enable the resection of large hemangiomas.

## Introduction

Liver hemangiomas are the most common benign liver tumors, with a prevalence of 5 to 20%. Most hemangiomas are small and require no treatment or only follow-up. However, giant hemangiomas, having a diameter of more than 4 cm or 5 cm, may give rise to mechanical complaints or coagulopathy requiring intervention [1]. The indication for treatment of giant liver hemangiomas remains a matter of debate; hemangiomas complicated with a consumptive coagulopathy (Kasabach-Merritt syndrome) or causing severe incapacitating symptoms are generally accepted as an absolute indication for surgical resection [2,3]. Once treatment is decided on, surgical excision is the most effective method with a low risk of morbidity and mortality [4,5], but other treatment options, including transcatheter arterial embolization (TAE) [6], and liver transplantation [7,8], are also sometimes advocated for large unresectable hemangiomas. Except for liver transplantation, however, palliative treatments usually do not produce satisfactory and sustained outcomes.

Here, we report a case of a single huge hemangioma, completely resected by a right trisectorectomy following two successive TAEs, by which the volume of the hemangioma was significantly reduced.

## Case presentation

A 38-year-old Asian man was referred to our hospital with complaints of severe abdominal distension and pancytopenia. His past or family medical history was unremarkable. Although his abdominal bloating was impairing his daily life, he had not visited a healthcare facility or undergone treatment for abdominal distension.

A routine blood count revealed pancytopenia, with a white blood cell count of 2600/μL, a red blood cell count of 3.42 × 10^6^/μL, a hemoglobin level of 10.3 g/dL, and a platelet count of 9.2 × 10^4^/μL. The results of liver function tests were normal, including a total bilirubin level of 1.3 mg/dL, an albumin level of 4.3 mg/dL, and an indocyanine green retention rate at 15 min of 8.7%. "Asymptomatic" Kasabach-Merritt syndrome was apparent, however, based on a high international normalized ratio of prothrombin time of 1.46, a decreased fibrinogen level of 82 mg/dL, elevated fibrin degradation products (FDP) of 80 μg/mL, and D-dimer levels of 32 μg/mL.

**Figure 1:**
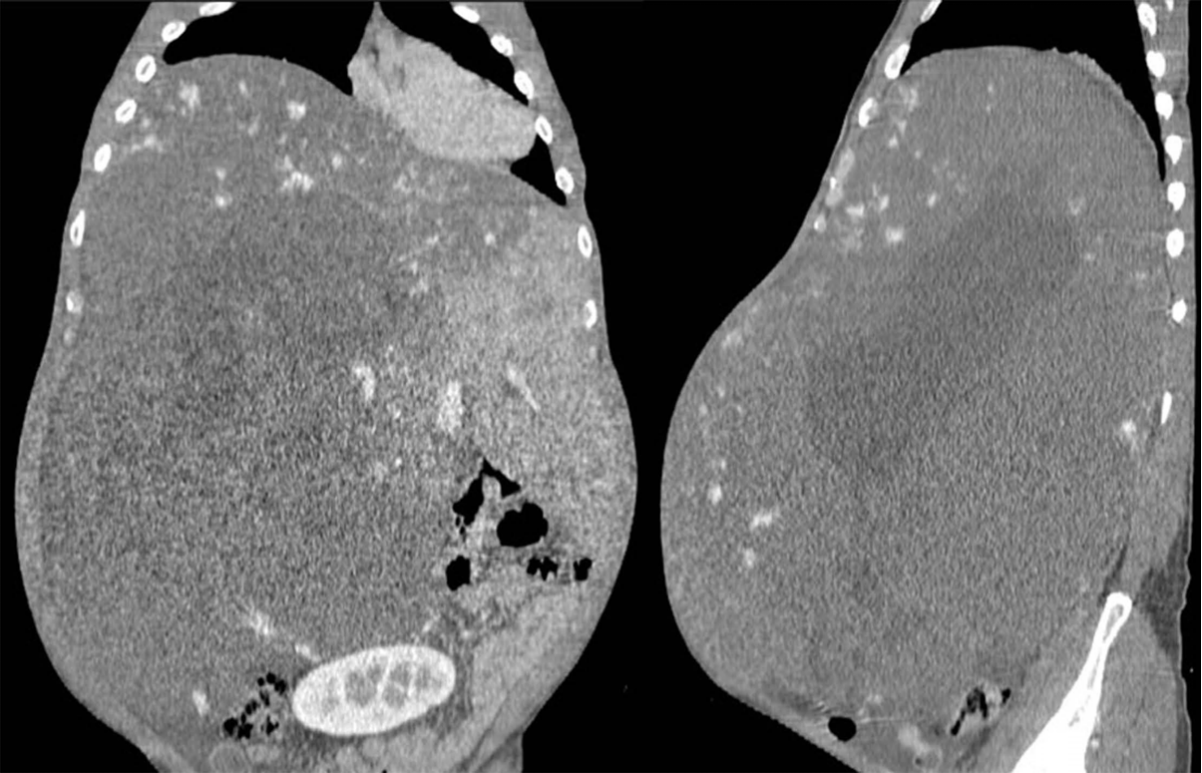
**Coronal and sagittal views of the hemangioma**. Reconstructed from multi-detector computed tomography images.

**Figure 2:**
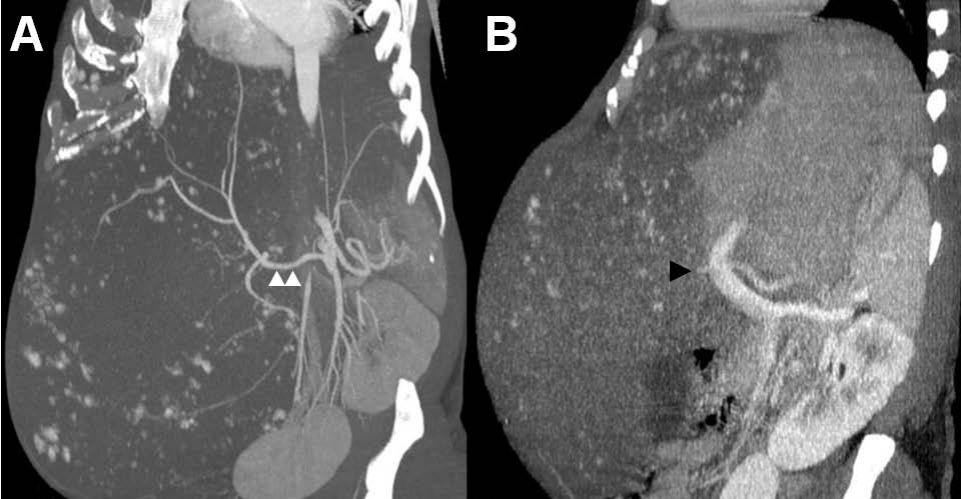
**Vascular reconstruction images**. **(**A) Angiography images reconstructed by multi-detector computed tomography (MDCT). The right hepatic artery (white arrowheads) and its branches are markedly stretched. (B) Portography images reconstructed by MDCT. The right portal vein is occluded (black arrowhead).

**Figure 3:**
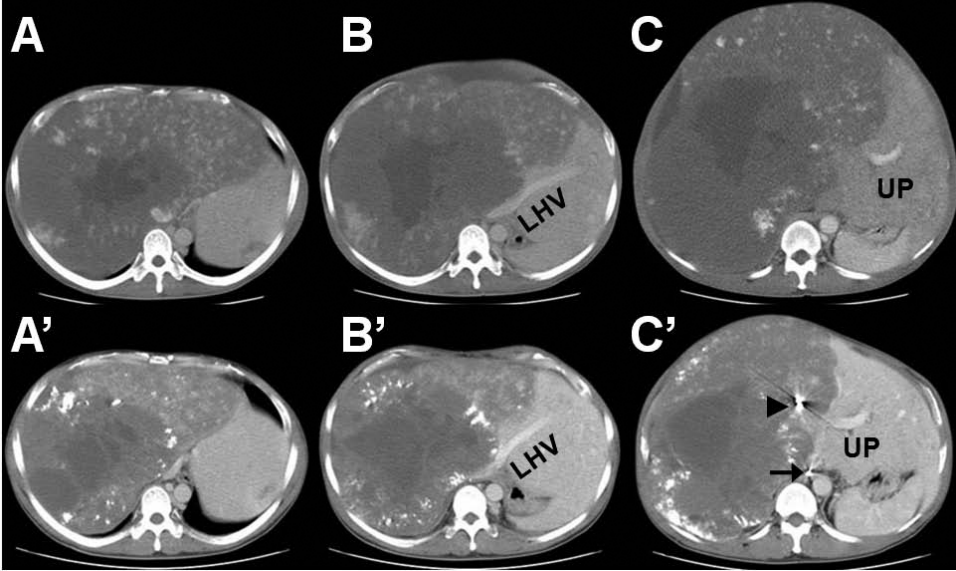
**Axial images of multi-detector computed tomography**. Multi-detector computed tomography (MDCT) images at the first visit (A-C), and corresponding MDCT slices just before the operation (i.e. after two sessions of transcatheter arterial embolization) (A'-C'). Metallic coils in the right hepatic artery (black arrowhead) and in the right sub-phrenic artery (black arrow) are indicated. LHV, left hepatic vein; UP, umbilical portion.

Multi-detector computed tomography (MDCT) on admission revealed a huge hemangioma located on the right liver, and replacing the parenchyma of the right liver and the left para-median sector. The hemangioma occupied almost the entire abdominal cavity, protruding into the right diaphragm up to the right thoracic cavity and extending down to the pelvic cavity, with a maximum diameter of 43 cm (Figure 1). Angiography and portography [reconstructed by MDCT images (Figure 2)] revealed that the right hepatic artery and its branches were extremely stretched, and the right portal vein was compressed and occluded by the tumor. The middle and right hepatic veins were completely occluded, and the hepatic vena cava was markedly compressed, while the left hepatic vein remained patent (Figure 3A-C). Volumetric analysis revealed a tumor volume of 16,880 mL and a left lateral sector volume of 1250 mL.

Based on the liver function tests, remnant liver volume, and anatomic considerations, urgent primary tumor resection seemed possible, but because of the benign nature of the disease and our patient's stable condition, we decided to perform TAE to reduce the tumor volume before performing the resection to ensure the safety of the future radical resection of the tumor.

First, TAE was performed for the right hepatic artery with coils and gelfoam. Thereafter, the tumor volume, anatomical positions, and recanalizations were calculated and investigated by dynamic MDCT once a month, not to misjudge the operation timing. Three months after the first TAE, the tumor volume had decreased to 10,290 mL. Angiography performed at that time revealed a collateral feeding artery from the right subphrenic artery, which was then embolized by TAE. Two months after the second TAE, the tumor volume had further decreased to 8260 mL based on MDCT volumetry (Figure 3A'-C'). No complication was observed after two successive TAEs.

**Figure 4:**
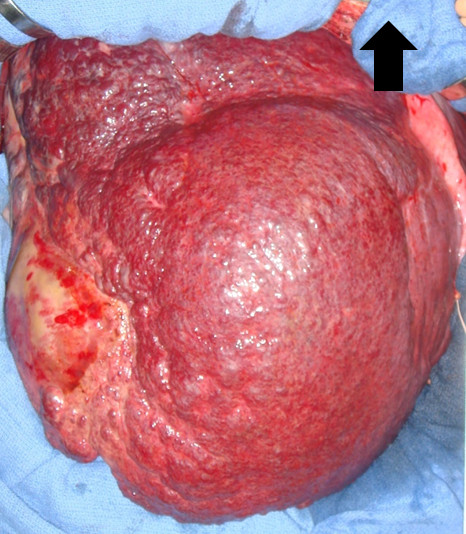
**Intra-operative photograph of the tumor**. Black arrow indicates the cranial side.

The remarkable volume reduction in the right upper portion of the tumor allowed for a safe approach to the hepatic veins and vena cava, and a radical resection of the tumor was performed by an anatomical right trisectorectomy. The operation was performed through a J-shaped skin incision using a ninth inter-costal thoracoabdominal approach (Figure 4), and after the conventional right liver mobilization, the tumor was successfully resected by anatomical division of the hepatic hilum preserving biliary continuity with the intermittent inflow occlusion. The operating time and the intra-operative blood loss were 540 min and 2150 mL, respectively.

Pathologic investigation of the specimen revealed a cavernous hemangioma, 30 × 25 × 15 cm, weighing 8100 g, comprising a spongy zone and a fibrotic scar zone with massive necrosis. Our patient's post-operative course was uneventful and he was discharged from the hospital 16 days after surgery. At 24 months following surgery, he enjoys an improved quality of life with normal liver function.

**Figure 5:**
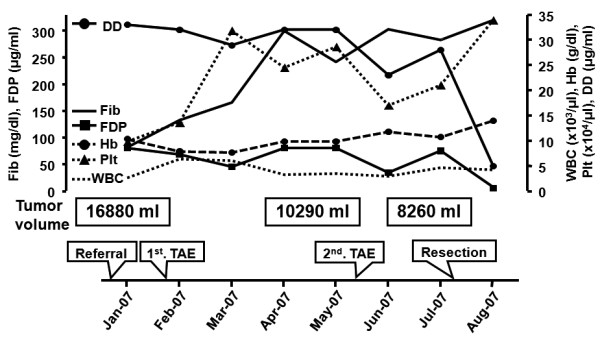
**Changes in the laboratory data and tumor volume**. WBC, white blood cell; Hb, hemoglobin; Plt, platelet; DD, D-dimer; Fib, fibrinogen; FDP, fibrin degradation products; TAE, transcatheter arterial embolization.

Changes in the laboratory data and tumor volume are summarized in Figure 5. Despite the blood counts becoming normal after TAE, fibrin degradation products (FDP) and D-dimer decreased after the surgical removal of the tumor.

## Discussion

Of the various treatment options for giant hemangiomas, surgical treatment, including resection and enucleation, provides the only consistently effective outcome with satisfactory results [4,5]. Although some authors reported that symptomatic giant liver hemangiomas can be managed successfully and non-invasively by TAE with a satisfactory decrease in symptoms and tumor volume [6], the effect of TAE generally seems to be variable and sometimes even results in a volume increase [7,8].

A recent major argument in the treatment of liver hemangiomas is the selection criteria for surgery; i.e. observation or operation, and enucleation or resection. Considering the benign and non-progressive nature of the disease, it is currently accepted that a giant hemangioma is not necessarily an indication for surgery just because of its size, and continued observation in asymptomatic patients or patients with minimal abdominal symptoms seems to be justified [2,3]. Because of variations in size, location, and number of tumors, the surgical strategy should be decided on a case-by-case basis. There seems to be general agreement that enucleation is better than resection in terms of sparing the liver parenchyma and decreasing intra-operative blood loss [4,5]. Large hemangiomas with severe incapacitating symptoms, such as in our case, or with symptomatic Kasabach-Merritt syndrome, however, are absolute indications for intervention, including surgical resection.

Hemostasis is important for resection of a giant hemangioma [5]. The larger the size and the greater the number of tumors, the more difficult it is to achieve hemostasis. Huge hemangiomas, multiple giant hemangiomas, and hemangiomatosis frequently require challenging operations, including extra-corporeal hepatic resection [9], hepatic resection with extra-corporeal circulation [10], or liver transplantation [7,8]. A review of the literature published over the last decade revealed several case reports in which these successful treatment options were used, but the documented blood loss (10,000 to 18,000 mL) during surgery might preclude these options from becoming standard treatment. Additionally, liver transplantation imposes life-long immunosuppression and the associated risks of complications.

In the present case, we considered that an urgent resection at the first admission would be dangerous because it seemed impossible to approach the confluence of hepatic veins and inferior vena cava behind the tumor without extra-corporeal circulation. Liver transplantation was also considered an option, but based on the benign nature of this disease and the patient's stable condition, we considered these aggressive options a last resort and decided to first perform TAE to reduce the tumor volume. The timing for the radical surgery after TAE is another problem in the management of huge liver hemangioma. Considering the vascular recanalization and various complications after TAE which might result in the loss of opportunity or the difficulty of the radical resection, some authors recommend urgent operation after TAE [11,12]. Fortunately, two successive TAEs with close monitoring by MDCT expanding across five months induced a significant volume reduction without any complication, enabling us to perform a safe and formulaic liver resection for this extraordinary tumor, however, when to operate should be decided case by case with meticulous investigations by surgeons not to misjudge the appropriate timing for the radical surgery.

## Conclusions

We report a case of a huge hemangioma, one of the largest tumors ever reported, that was successfully resected following effective TAE. The results in our case indicate the importance of pre-operative management to reduce tumor size.

## Abbreviations

FDP: fibrin degradation products; MDCT: multi-detector computed tomography; TAE: transcatheter arterial embolization.

## Consent

Written informed consent was obtained from the patient for publication of this case report and any accompanying images. A copy of the written consent is available for review by the Editor-in-Chief of this journal.

## Competing interests

The authors declare that they have no competing interests.

## Authors' contributions

AN was responsible for the management of this case. AN and SY were major contributors in writing the manuscript. All authors read and approved the final manuscript.
